# Cerebral amyloid angiopathy mimicking central nervous system metastases: a case report

**DOI:** 10.1186/s13256-018-1655-6

**Published:** 2018-05-14

**Authors:** Christopher DeZorzi, Ruth Fernandez-Ruiz, Sarika Gupta, Katherine Harris

**Affiliations:** University of Iowa Hospitals and Clinics, 200 Hawkins Dr, Iowa City, IA 52242 USA

**Keywords:** Clinical pathology, Cerebral amyloid angiopathy, Dementia, Intracranial hemorrhage, Alzheimer’s disease

## Abstract

**Background:**

This case describes an unusual presentation of an intracranial hemorrhage first thought to be metastatic disease on computed tomography and magnetic resonance imaging. The healthcare team completed an exhaustive search for a primary malignancy that was negative. Final diagnosis on brain biopsy showed intercranial hemorrhage secondary to cerebral amyloid angiopathy. With an increasing number of elderly patients and the rising cost of health care, this case can serve as a reminder to clinicians about their own responsibilities in limiting the cost of health care.

**Case presentation:**

This is a case report about a 72-year-old white woman with an intracranial hemorrhage secondary to cerebral amyloid angiopathy. The brain lesions on computed tomography/magnetic resonance imaging mimicked a metastatic process until a brain biopsy could give a definitive diagnosis that was completely unexpected. Cerebral amyloid angiopathy is a rare cause of intracerebral hemorrhage and this diagnosis is important to consider in older patients on anticoagulation.

**Conclusions:**

Cerebral amyloid angiopathy is a rare diagnosis but should be considered in elderly patients on anticoagulation presenting with imaging findings consistent with intracerebral hemorrhage. While metastatic disease is a more common cause of intracerebral hemorrhage, cerebral amyloid angiopathy should remain in the differential diagnosis. This case report serves as a teaching point to clinicians in cases involving an older patient on anticoagulation.

## Background

Cerebral amyloid angiopathy (CAA) is characterized by amyloid deposition in cerebral blood vessels. This deposition consists of β-amyloid peptide in the media of small and medium-sized cerebral arteries. Marked deposition can lead to necrosis of the vessel walls and hemorrhage. It is commonly associated with Alzheimer’s disease (AD) but most patients with hemorrhage related to CAA do not have AD [1]. It can occur as a sporadic disorder, sometimes in association with AD, or as a familial syndrome. CAA is a cause of spontaneous cerebral hemorrhage especially in the elderly and may present as a sudden unexpected death in an older person. Patients on orally administered anticoagulation with CAA are also especially prone to intracranial hemorrhage (ICH). Many of our older patient population on orally administered anticoagulation would be at high risk of bleeding if they had CAA. The definitive diagnosis of this condition still requires pathological examination of brain tissue. The prevalence of CAA is markedly age dependent. It can be identified pathologically in up to 35% of brains from individuals aged 85 years or older. One-third of these older patients with CAA are affected to the extent associated with hemorrhage [2, 3]. In younger patients, the prevalence of moderate to severe CAA is 2.3% for patients between the ages of 65 and 74 and 8.0% between the ages of 75 and 84 [3]. Although hypertensive vasculopathy is the leading cause of ICH in the deep hemispheric regions of the brain, CAA may be more common for lobar ICH [4]. This is a cause for concern, considering the lack of treatment options we have for CAA compared with hypertensive vasculopathy.

## Case presentation

**Fig. 1:**
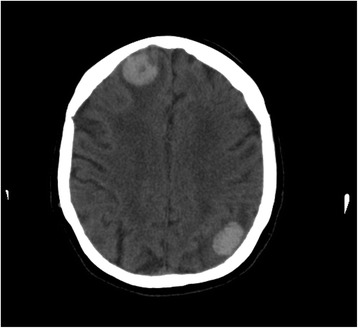
Computed tomography scan of the brain showed multiple areas of hemorrhage

**Fig. 2:**
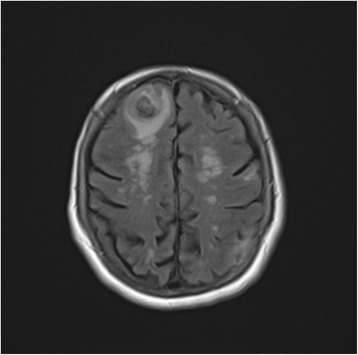
Magnetic resonance imaging of the brain was remarkable for multiple hemorrhagic ring-enhancing lesions with surrounding vasogenic edema, highly suspicious for hemorrhagic metastases versus abscesses

**Fig. 3:**
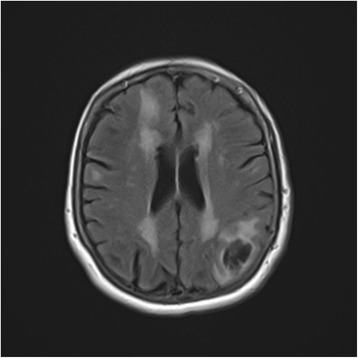
Magnetic resonance imaging of the brain was remarkable for multiple hemorrhagic ring-enhancing lesions with surrounding vasogenic edema, highly suspicious for hemorrhagic metastases versus abscesses

**Fig. 4:**
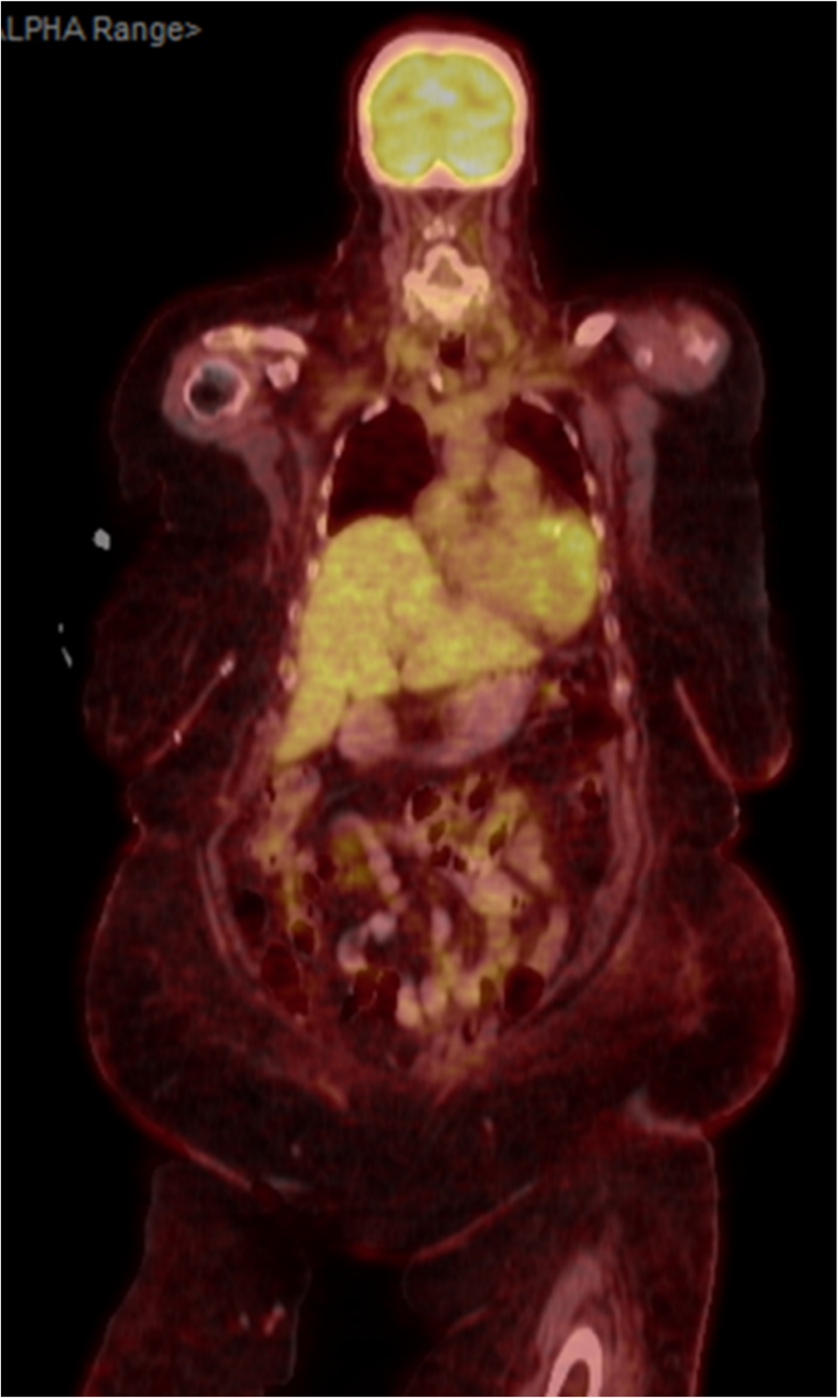
Positron emission tomography scan was negative as a part of workup to look for a primary lesion

**Fig. 5:**
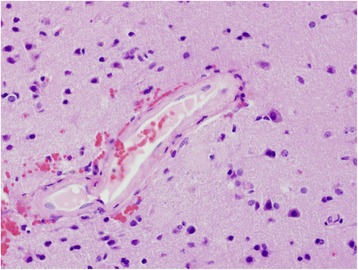
Hematoxylin and eosin stained section (× 40) illustrating hyalinization of intra-parenchymal arterioles

**Fig. 6:**
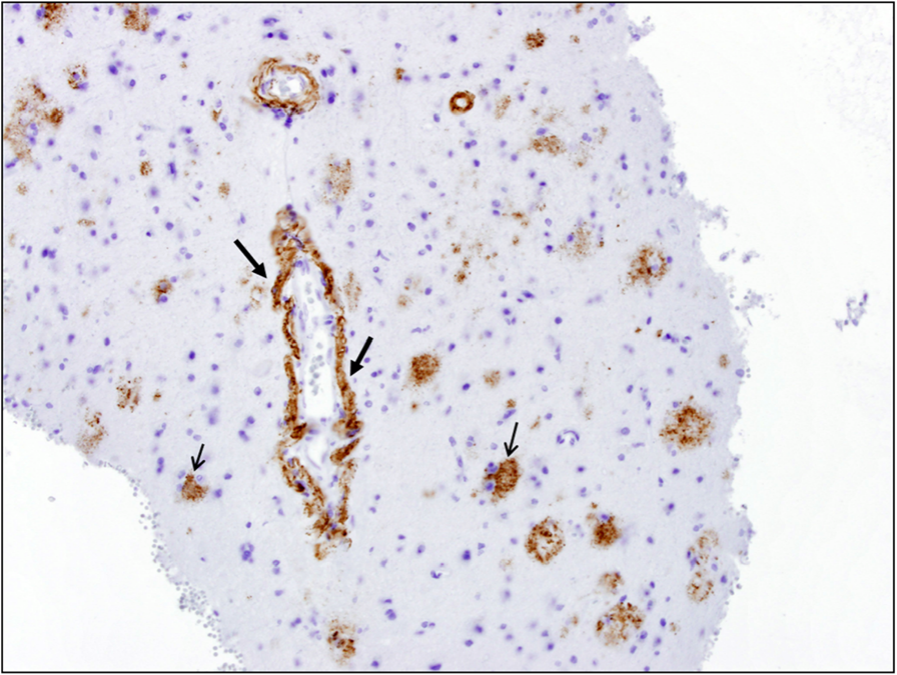
Immunohistochemical staining (× 20) highlights the amyloid beta deposition in vessel walls (*thick arrows*). Scattered amyloid plaques are also present (*thin arrows*)

A 72-year-old white woman with a medical history of recurrent pneumonia, pulmonary embolism 1 month ago (currently on warfarin therapy), and coronary artery disease presented from an independent living facility with altered mentation. She had no known family history of CAA or ICH and had not been brought into the Emergency Room (ER) for confusion previously. At baseline, she was very independent, living on her own, and was able to perform all her activities of daily living (ADL). Her family indicated that she started to have worsening altered mental status, to the point of being found profoundly confused by staff in the lobby of her living facility, prompting her urgent transfer to a local hospital by the Emergency Medical Service. On presentation, she was febrile to 39.9 °C and had right-sided deficits with repetitive speech. A computed tomography (CT) scan (Fig. 1) was concerning for multiple areas of hemorrhage, therefore, she was transferred to our tertiary hospital. She arrived hemodynamically stable with no focal deficits on neurologic examination. Magnetic resonance imaging (MRI) of her brain showed multiple hemorrhagic ring-enhancing lesions with surrounding vasogenic edema, highly suspicious for hemorrhagic metastasis (Figs. 2 and 3). In the Medical Intensive Care Unit, she received 4-factor prothrombin complex concentrate and vitamin K to reverse her international normalized ratio (INR), broad-spectrum antibiotics for the possibility of the lesions being infectious, intravenously administered dexamethasone to reduce brain edema, and levetiracetam after an electroencephalogram (EEG) showed a seizure tendency. Over the next few days she had no signs or obvious source of infection, so an exhaustive workup in an attempt to find a primary malignancy was initiated. A CT of her chest, abdomen, and pelvis resulted in no findings suggestive of a primary lesion. A thyroid ultrasound, mammogram, and positron emission tomography (PET)/CT (skull base to upper thigh) were also negative (Fig. 4). Further evaluation with dermatology showed basal cell carcinoma of the nasal wall, but no evidence of melanoma. Finally, the neurosurgery team was consulted to discuss options for tissue diagnosis and a brain tissue biopsy was obtained. The results showed amyloid angiopathy and an organized hematoma with gliosis but no signs of neoplasm (Figs. 5 and 6). Discussions among the primary care team and specialty teams then changed focus to anticoagulation due to her recent pulmonary embolism. Given her new diagnosis, she would be a very high-risk anticoagulation candidate due to her increased risk of intracerebral hemorrhage, so an inferior vena cava filter was placed. For discharge, she was prescribed a dexamethasone taper, continued on levetiracetam, scheduled for a follow-up MRI in 1 month, and was discharged to a skilled nursing facility with slightly increased confusion from her baseline but much improved from her presentation. On follow-up 1 year later she was showing increasing signs of dementia. She lives in an assisted living facility but maintains a majority of her ADL independently.

## Discussion

As the medical field has developed more advances and pharmaceutical options for hypertensive control, the expected decrease in the incidence of ICH has not followed. The overall incidence is actually stable, which could be secondary to orally administered anticoagulation-associated bleeds in our ageing population [5]. Warfarin is the most commonly used orally administered anticoagulant and works by inhibiting vitamin K-dependent coagulation factors II, VII, IX, and X. The anti-thromboembolic benefits of anticoagulation have been proven significantly useful in several cardiac-related conditions and for venous thromboembolisms even beyond its risk of bleeding [6]. The exact nature of orally administered anticoagulant-associated ICH is likely intertwined with the effects of ageing, genetics, and other concurrent vascular conditions on the brain. Older patients who have CAA in addition to anticoagulation will be especially prone to ICH.

Of interest, in this clinical scenario CT and MRI actually led the primary team toward chasing after the wrong underlying etiology of the ICH. The reports from radiology for both the CT and MRI stated that the lesions were most consistent with metastasis. After the primary team reviewed this with radiology, a complete and very expensive workup to find the primary lesion followed. This exhaustive workup creates a huge burden and contributes to the rising cost of health care. Although in this scenario it was appropriate to attempt to find primary malignant lesions, this case can be used as a reminder to keep in mind the possibility of ICH due to CAA especially in the presence of an older patient on anticoagulation. It is important to remember ICH is not always associated with hypertension or Alzheimer’s dementia.

Another topic in this clinical scenario is whether to restart the orally administered anticoagulation. There is an elevated risk of CAA and ICH in the elderly, which is a given reason to hold anticoagulation. This is counterbalanced by a parallel increased risk of thromboembolic stroke with increasing age [2]. The benefits of stroke prevention, or prevention of pulmonary embolism in this case, many times outweigh the risks of bleeding in patients when deciding to start or continue anticoagulation. In this case, it was decided that anticoagulation risks posed a larger problem than our patient’s previous pulmonary embolism. Given the large role of CAA as an underlying cause of anticoagulant-related ICH [7] and the high rate of ICH recurrence in patients with CAA [8], it is recommended that long-term anticoagulation not be prescribed for survivors of suspected CAA-related ICH who have non-valvular atrial fibrillation [5, 9]. It is unknown, but likely, that the same risks would outweigh the benefits in a patient with a history of pulmonary embolism.

## Conclusions

- CAA is a rare cause of intracerebral hemorrhage
- This diagnosis is important to consider in older patients on anticoagulation presenting with ICH
- A lack of hypertension can be a clue to CAA causing ICH
- The use of anticoagulants increases the risk of brain hemorrhage in patients with CAA
- With the knowledge of this case report, clinicians should now be able to devise broader differential diagnoses in similar situations
